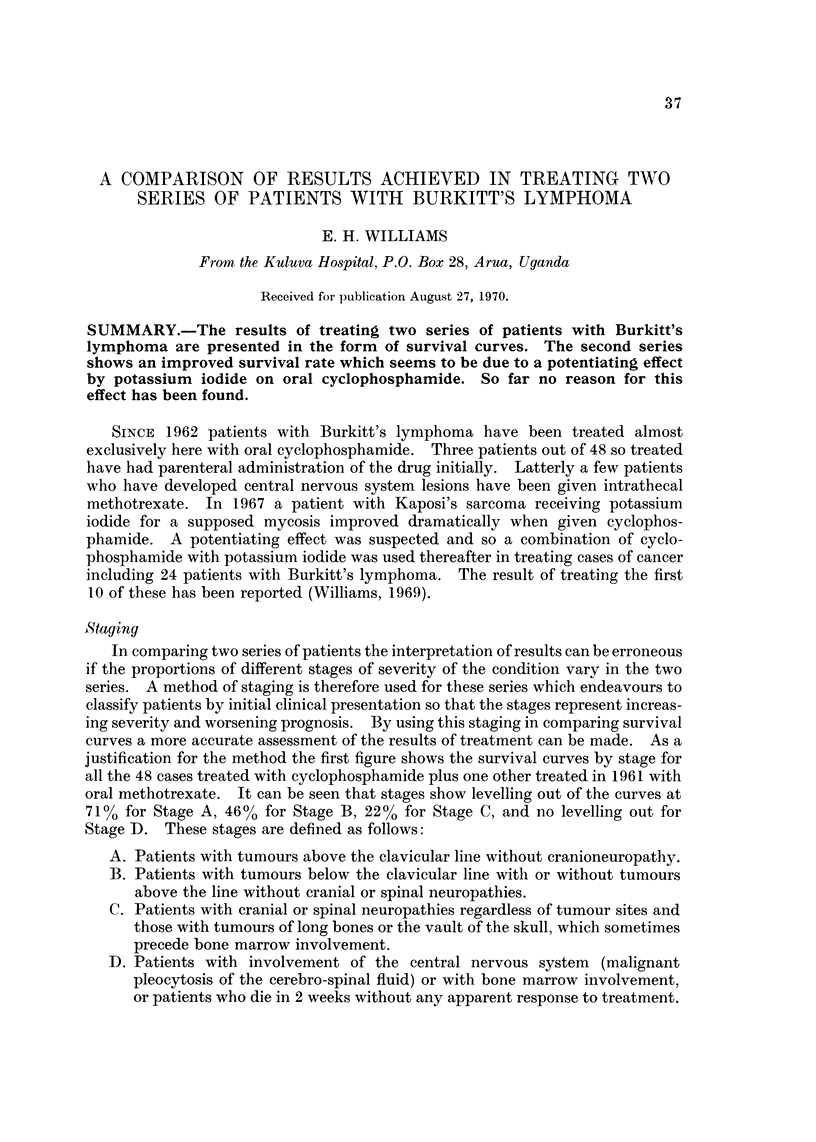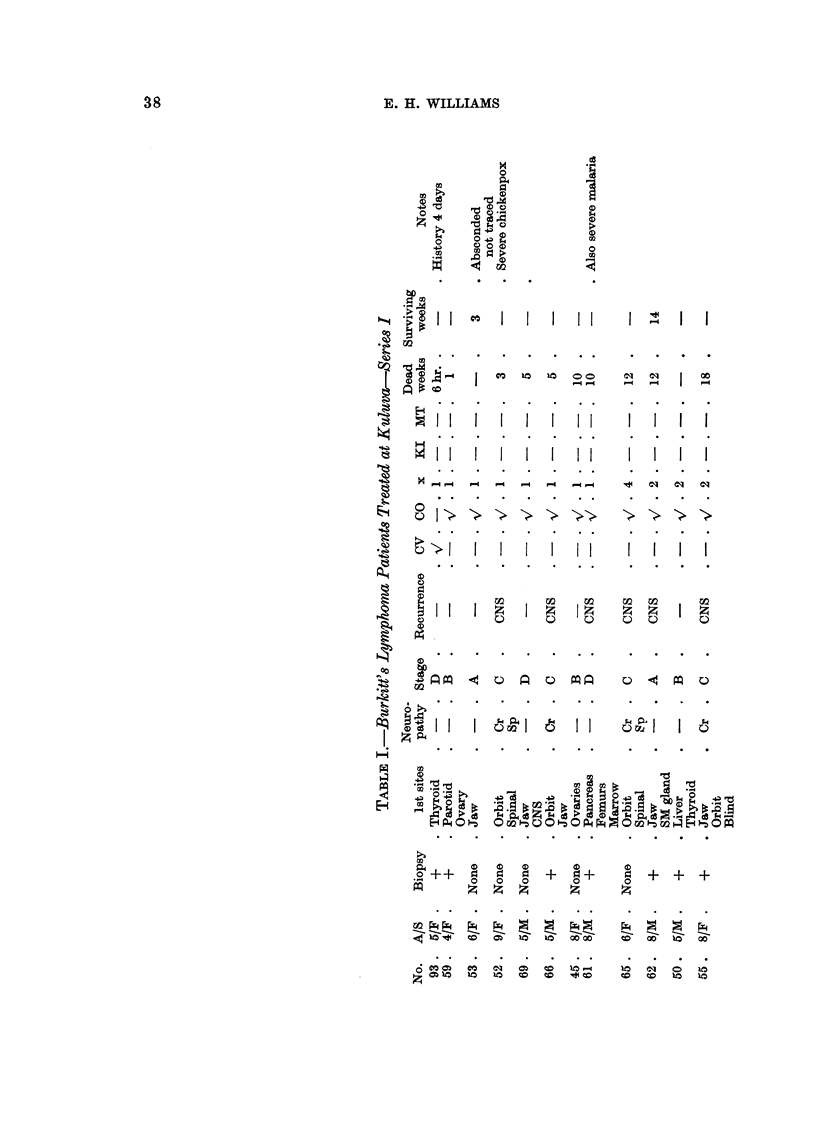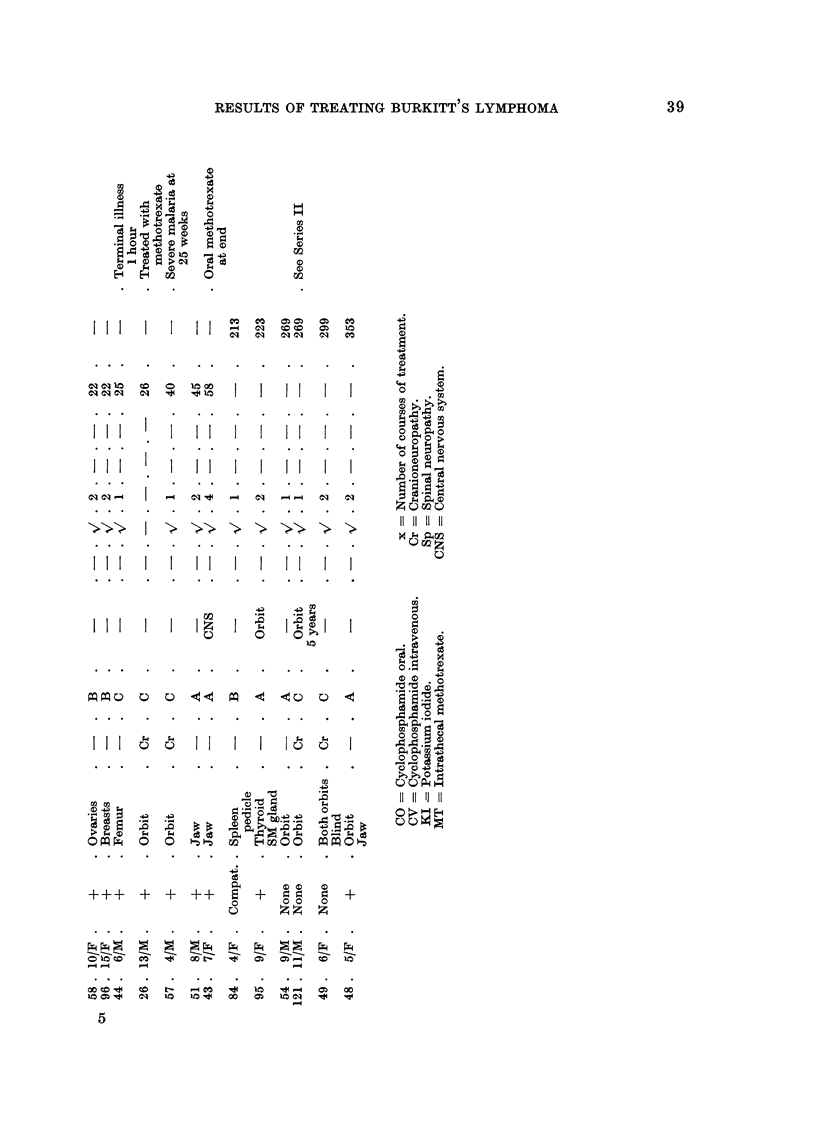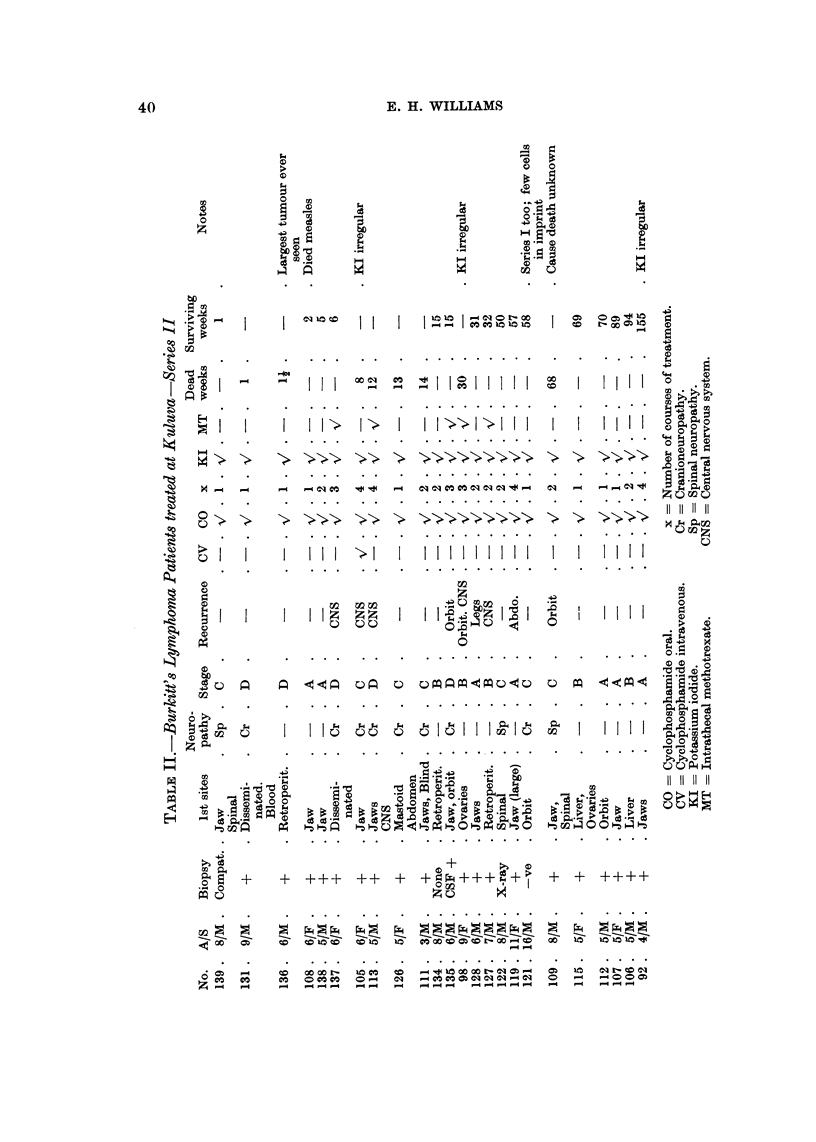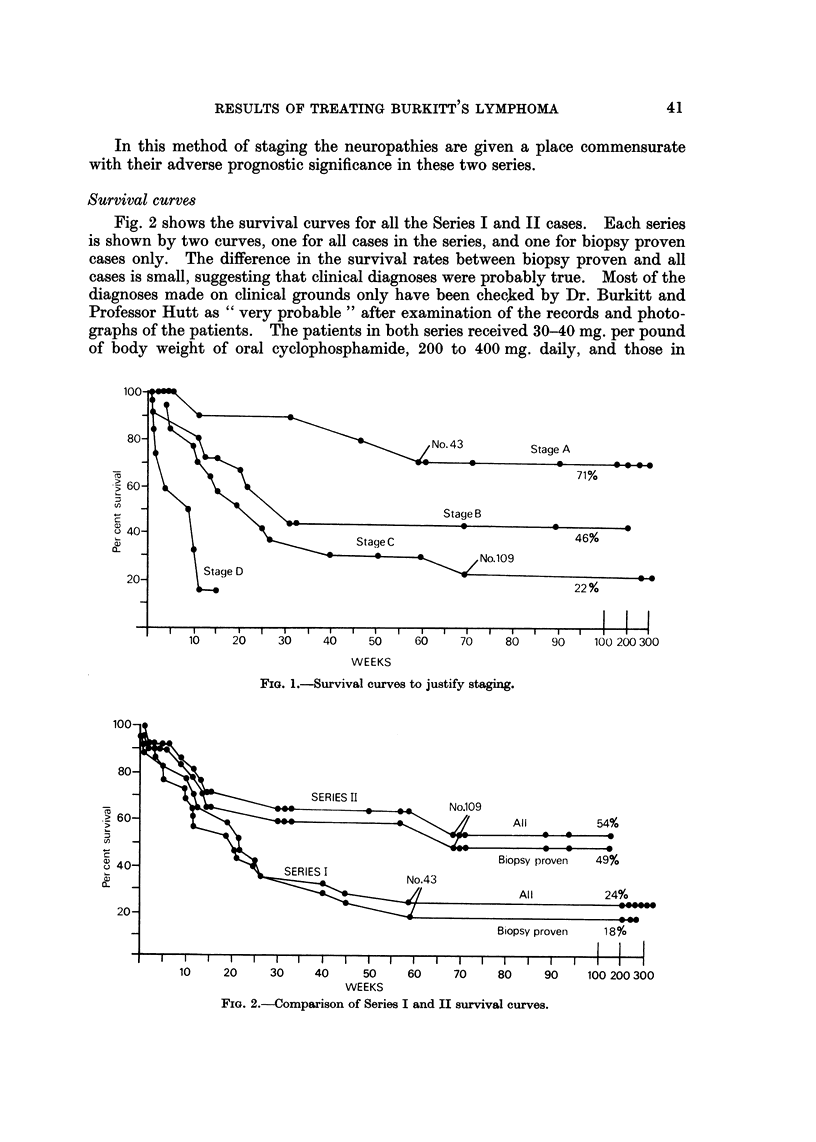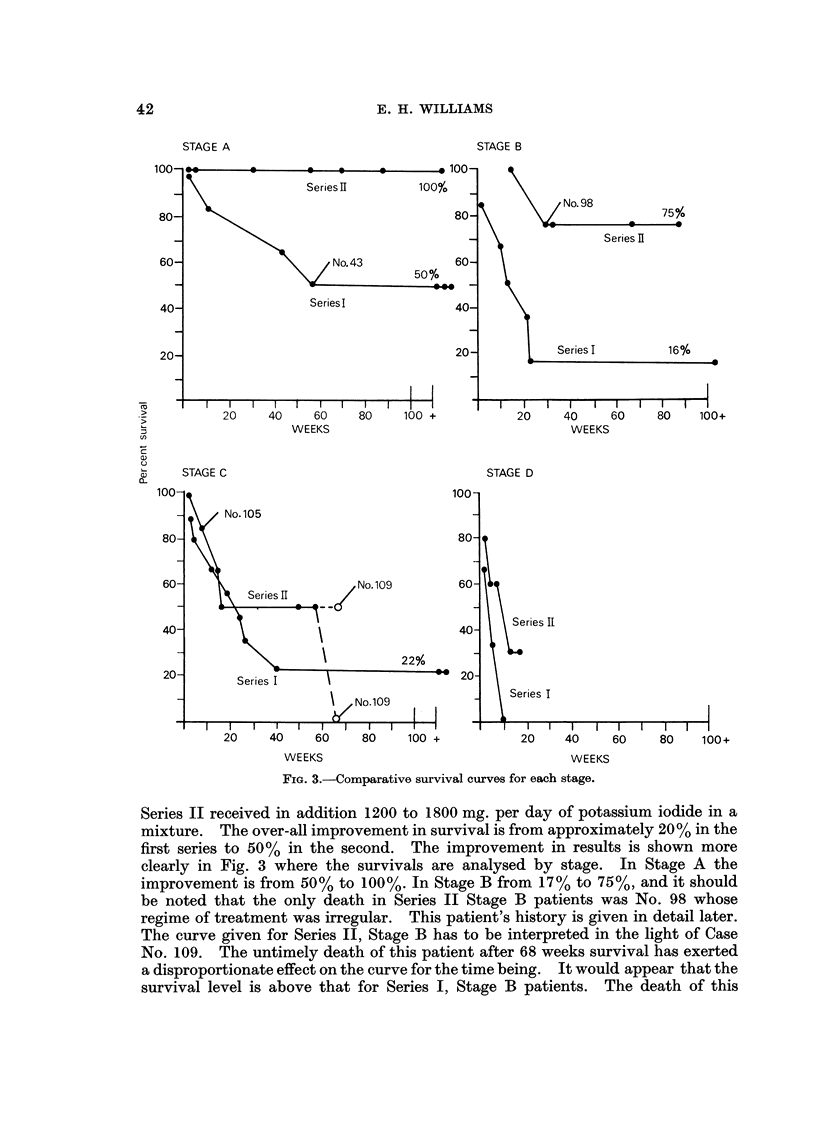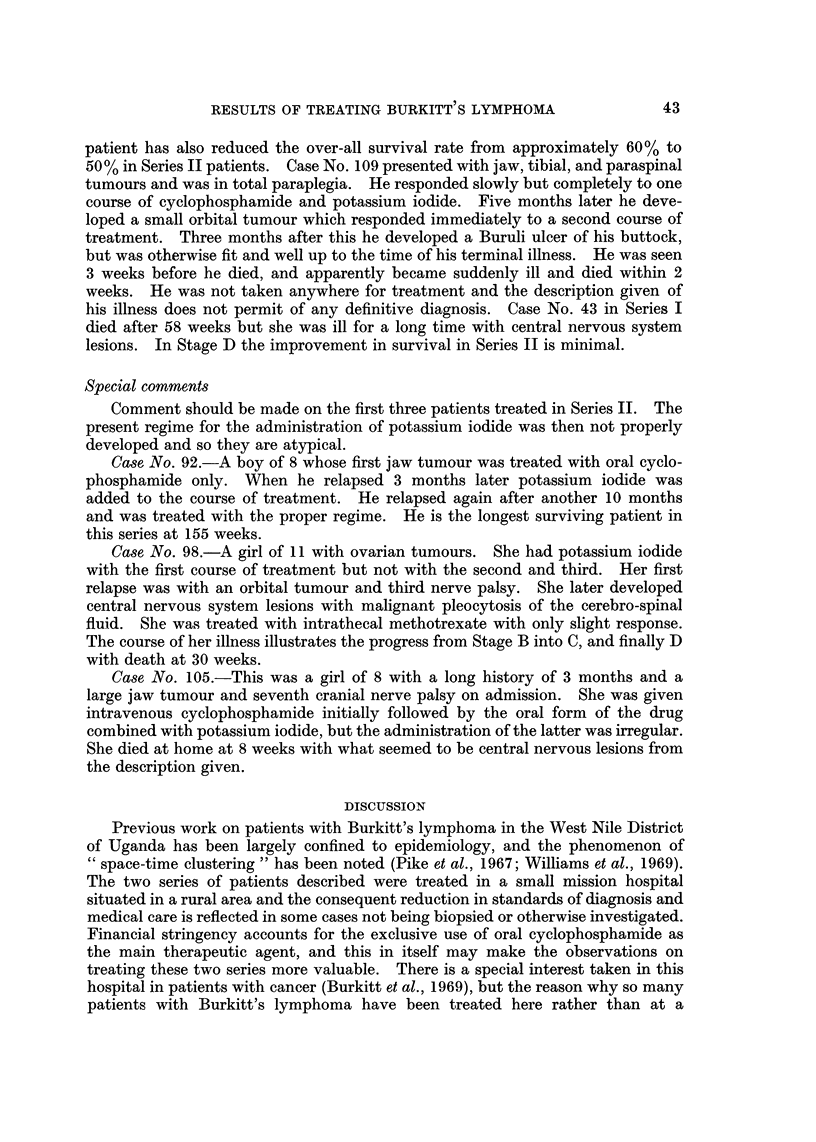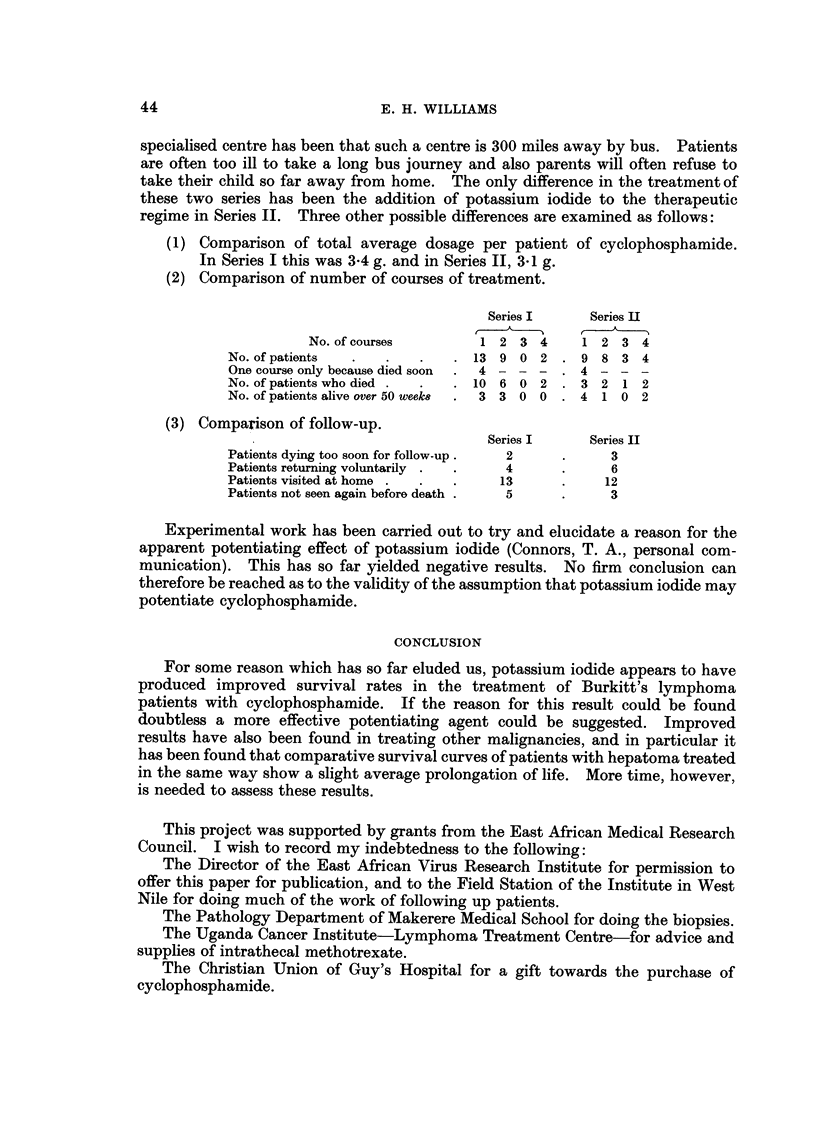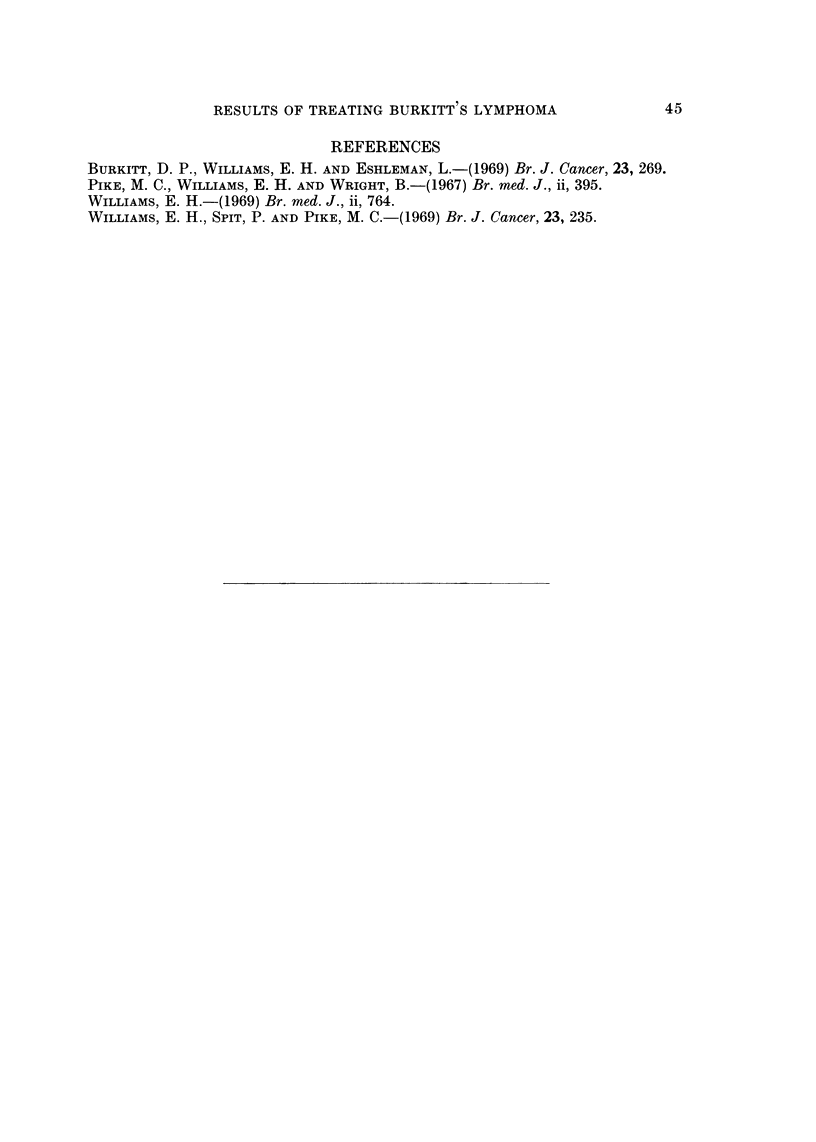# A Comparison of Results Achieved in Treating Two Series of Patients with Burkitt's Lymphoma

**DOI:** 10.1038/bjc.1971.6

**Published:** 1971-03

**Authors:** E. H. Williams

## Abstract

The results of treating two series of patients with Burkitt's lymphoma are presented in the form of survival curves. The second series shows an improved survival rate which seems to be due to a potentiating effect by potassium iodide on oral cyclophosphamide. So far no reason for this effect has been found.


### A COMPARISON OF RESULTS ACHIEVED IN TREATING TWO SERIES OF PATIENTS WITH BURKITT'S LYMPHOMA

E. H. WILLIAMS

*From the Kuluva Hospital, P.O. Box 28, Arua, Uganda*

Received for publication August 27, 1970.

**SUMMARY.—The results of treating two series of patients with Burkitt's lymphoma are presented in the form of survival curves. The second series shows an improved survival rate which seems to be due to a potentiating effect by potassium iodide on oral cyclophosphamide. So far no reason for this effect has been found.**

SINCE 1962 patients with Burkitt's lymphoma have been treated almost exclusively here with oral cyclophosphamide. Three patients out of 48 so treated have had parenteral administration of the drug initially. Latterly a few patients who have developed central nervous system lesions have been given intrathecal methotrexate. In 1967 a patient with Kaposi's sarcoma receiving potassium iodide for a supposed mycosis improved dramatically when given cyclophosphamide. A potentiating effect was suspected and so a combination of cyclophosphamide with potassium iodide was used thereafter in treating cases of cancer including 24 patients with Burkitt's lymphoma. The result of treating the first 10 of these has been reported (Williams, 1969).

*Staging*

In comparing two series of patients the interpretation of results can be erroneous if the proportions of different stages of severity of the condition vary in the two series. A method of staging is therefore used for these series which endeavours to classify patients by initial clinical presentation so that the stages represent increasing severity and worsening prognosis. By using this staging in comparing survival curves a more accurate assessment of the results of treatment can be made. As a justification for the method the first figure shows the survival curves by stage for all the 48 cases treated with cyclophosphamide plus one other treated in 1961 with oral methotrexate. It can be seen that stages show levelling out of the curves at 71% for Stage A, 46% for Stage B, 22% for Stage C, and no levelling out for Stage D. These stages are defined as follows:

A. Patients with tumours above the clavicular line without cranioneuropathy.
B. Patients with tumours below the clavicular line with or without tumours above the line without cranial or spinal neuropathies.
C. Patients with cranial or spinal neuropathies regardless of tumour sites and those with tumours of long bones or the vault of the skull, which sometimes precede bone marrow involvement.
D. Patients with involvement of the central nervous system (malignant pleocytosis of the cerebro-spinal fluid) or with bone marrow involvement, or patients who die in 2 weeks without any apparent response to treatment.

x = Number of courses of treatment.

In this method of staging the neuropathies are given a place commensurate with their adverse prognostic significance in these two series.

*Survival curves*

Fig. 2 shows the survival curves for all the Series I and II cases. Each series is shown by two curves, one for all cases in the series, and one for biopsy proven cases only. The difference in the survival rates between biopsy proven and all cases is small, suggesting that clinical diagnoses were probably true. Most of the diagnoses made on clinical grounds only have been checked by Dr. Burkitt and Professor Hutt as " very probable " after examination of the records and photographs of the patients. The patients in both series received 30–40 mg. per pound of body weight of oral cyclophosphamide, 200 to 400 mg. daily, and those in

FIG. 1.—Survival curves to justify staging.

FIG. 2.—Comparison of Series I and II survival curves.

FIG. 3.—Comparative survival curves for each stage.

Series II received in addition 1200 to 1800 mg. per day of potassium iodide in a mixture. The over-all improvement in survival is from approximately 20% in the first series to 50% in the second. The improvement in results is shown more clearly in Fig. 3 where the survivals are analysed by stage. In Stage A the improvement is from 50% to 100%. In Stage B from 17% to 75%, and it should be noted that the only death in Series II Stage B patients was No. 98 whose regime of treatment was irregular. This patient's history is given in detail later. The curve given for Series II, Stage B has to be interpreted in the light of Case No. 109. The untimely death of this patient after 68 weeks survival has exerted a disproportionate effect on the curve for the time being. It would appear that the survival level is above that for Series I, Stage B patients. The death of this patient has also reduced the over-all survival rate from approximately 60% to 50% in Series II patients. Case No. 109 presented with jaw, tibial, and paraspinal tumours and was in total paraplegia. He responded slowly but completely to one course of cyclophosphamide and potassium iodide. Five months later he developed a small orbital tumour which responded immediately to a second course of treatment. Three months after this he developed a Buruli ulcer of his buttock, but was otherwise fit and well up to the time of his terminal illness. He was seen 3 weeks before he died, and apparently became suddenly ill and died within 2 weeks. He was not taken anywhere for treatment and the description given of his illness does not permit of any definitive diagnosis. Case No. 43 in Series I died after 58 weeks but she was ill for a long time with central nervous system lesions. In Stage D the improvement in survival in Series II is minimal.

*Special comments*

Comment should be made on the first three patients treated in Series II. The present regime for the administration of potassium iodide was then not properly developed and so they are atypical.

*Case No.* 92.—A boy of 8 whose first jaw tumour was treated with oral cyclophosphamide only. When he relapsed 3 months later potassium iodide was added to the course of treatment. He relapsed again after another 10 months and was treated with the proper regime. He is the longest surviving patient in this series at 155 weeks.

*Case No.* 98.—A girl of 11 with ovarian tumours. She had potassium iodide with the first course of treatment but not with the second and third. Her first relapse was with an orbital tumour and third nerve palsy. She later developed central nervous system lesions with malignant pleocytosis of the cerebro-spinal fluid. She was treated with intrathecal methotrexate with only slight response. The course of her illness illustrates the progress from Stage B into C, and finally D with death at 30 weeks.

*Case No.* 105.—This was a girl of 8 with a long history of 3 months and a large jaw tumour and seventh cranial nerve palsy on admission. She was given intravenous cyclophosphamide initially followed by the oral form of the drug combined with potassium iodide, but the administration of the latter was irregular. She died at home at 8 weeks with what seemed to be central nervous lesions from the description given.

### DISCUSSION

Previous work on patients with Burkitt's lymphoma in the West Nile District of Uganda has been largely confined to epidemiology, and the phenomenon of " space-time clustering " has been noted (Pike *et al.*, 1967; Williams *et al.*, 1969). The two series of patients described were treated in a small mission hospital situated in a rural area and the consequent reduction in standards of diagnosis and medical care is reflected in some cases not being biopsied or otherwise investigated. Financial stringency accounts for the exclusive use of oral cyclophosphamide as the main therapeutic agent, and this in itself may make the observations on treating these two series more valuable. There is a special interest taken in this hospital in patients with cancer (Burkitt *et al.*, 1969), but the reason why so many patients with Burkitt's lymphoma have been treated here rather than at a specialised centre has been that such a centre is 300 miles away by bus. Patients are often too ill to take a long bus journey and also parents will often refuse to take their child so far away from home. The only difference in the treatment of these two series has been the addition of potassium iodide to the therapeutic regime in Series II. Three other possible differences are examined as follows:

(1) Comparison of total average dosage per patient of cyclophosphamide. In Series I this was 3·4 g. and in Series II, 3·1 g.
(2) Comparison of number of courses of treatment.

| | Series I | | | | Series II | | | |
|---|---|---|---|---|---|---|---|---|
| No. of courses | 1 | 2 | 3 | 4 | 1 | 2 | 3 | 4 |
| No. of patients | 13 | 9 | 0 | 2 | 9 | 8 | 3 | 4 |
| One course only because died soon | 4 | – | – | – | 4 | – | – | – |
| No. of patients who died | 10 | 6 | 0 | 2 | 3 | 2 | 1 | 2 |
| No. of patients alive *over* 50 *weeks* | 3 | 3 | 0 | 0 | 4 | 1 | 0 | 2 |

(3) Comparison of follow-up.

| | Series I | Series II |
|---|---|---|
| Patients dying too soon for follow-up | 2 | 3 |
| Patients returning voluntarily | 4 | 6 |
| Patients visited at home | 13 | 12 |
| Patients not seen again before death | 5 | 3 |

Experimental work has been carried out to try and elucidate a reason for the apparent potentiating effect of potassium iodide (Connors, T. A., personal communication). This has so far yielded negative results. No firm conclusion can therefore be reached as to the validity of the assumption that potassium iodide may potentiate cyclophosphamide.

### CONCLUSION

For some reason which has so far eluded us, potassium iodide appears to have produced improved survival rates in the treatment of Burkitt's lymphoma patients with cyclophosphamide. If the reason for this result could be found doubtless a more effective potentiating agent could be suggested. Improved results have also been found in treating other malignancies, and in particular it has been found that comparative survival curves of patients with hepatoma treated in the same way show a slight average prolongation of life. More time, however, is needed to assess these results.

This project was supported by grants from the East African Medical Research Council. I wish to record my indebtedness to the following:

The Director of the East African Virus Research Institute for permission to offer this paper for publication, and to the Field Station of the Institute in West Nile for doing much of the work of following up patients.

The Pathology Department of Makerere Medical School for doing the biopsies.

The Uganda Cancer Institute—Lymphoma Treatment Centre—for advice and supplies of intrathecal methotrexate.

The Christian Union of Guy's Hospital for a gift towards the purchase of cyclophosphamide.

### REFERENCES

BURKITT, D. P., WILLIAMS, E. H. AND ESHLEMAN, L.—(1969) *Br. J. Cancer*, **23**, 269.
PIKE, M. C., WILLIAMS, E. H. AND WRIGHT, B.—(1967) *Br. med. J.*, ii, 395.
WILLIAMS, E. H.—(1969) *Br. med. J.*, ii, 764.
WILLIAMS, E. H., SPIT, P. AND PIKE, M. C.—(1969) *Br. J. Cancer*, **23**, 235.